# A dual space MRI radiomic network signature for risk stratification and subtyping of mild cognitive impairment

**DOI:** 10.1016/j.isci.2026.116394

**Published:** 2026-06-12

**Authors:** Diaohan Xiong, Mengjiao Liu, Jiamei Xie, Zefeng Liu, Junping Wang

**Affiliations:** 1 Department of Radiology, Tianjin Key Lab of Functional Imaging & Tianjin Institute of Radiology, Tianjin Medical University General Hospital, Tianjin 300052, China

**Keywords:** applied sciences, biological sciences, cognitive neuroscience, computer science, health sciences, medical imaging, medical tests, medicine, natural sciences, network, neuroscience

## Abstract

Mild cognitive impairment (MCI) is a clinically heterogeneous prodromal stage of Alzheimer’s disease (AD) in which accurate risk stratification could support earlier intervention and more efficient trial design. Using baseline T1-weighted MRI from the Alzheimer’s Disease Neuroimaging Initiative and the National Alzheimer’s Coordinating Center cohorts, we developed a dual-space anatomical radiomic-network signature that integrates regional radiomic features and radiomics similarity network metrics extracted in standard and native spaces. The signature improved prediction of MCI-to-AD conversion relative to regional volumetric and clinical models, achieved consistent external validation performance, and separated participants into high- and low-risk groups. The same feature set identified five imaging-defined MCI subtypes with distinct anatomical patterns, conversion risks, and clinical, APOE ε4, and cerebrospinal fluid biomarker profiles; fronto-parietal and parahippocampal-temporal subtypes showed the highest risk. These findings support structural MRI radiomic-network profiling as a complementary framework for individualized prognosis and cohort stratification in AD research.

## Highlights

• Dual-space MRI radiomic-network signature predicts MCI conversion
• ARN-Sig outperforms volumetric and clinical models in validation
• Five MRI-defined MCI subtypes show distinct conversion risks
• High-risk subtypes show AD-consistent clinical and biomarker profiles

## Graphical abstract

**Figure:**
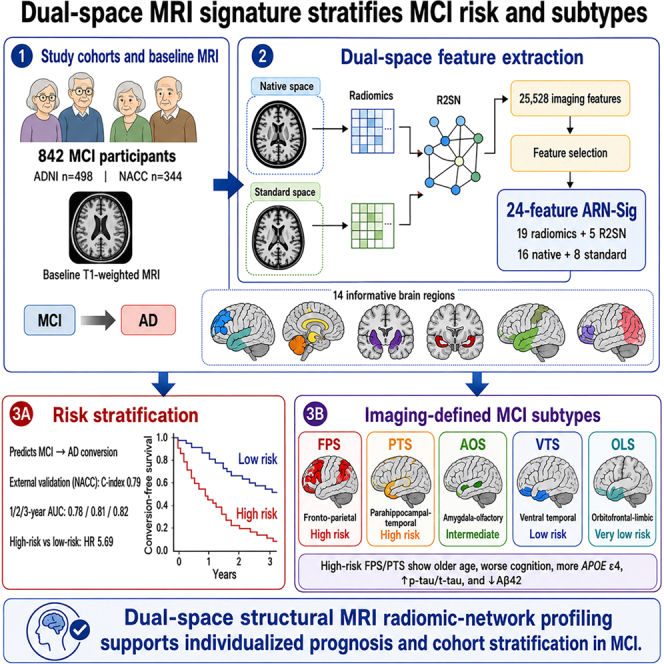

## Introduction

Mild cognitive impairment (MCI), often representing the prodromal stage of Alzheimer’s disease (AD), is a critical transitional state between normal cognitive aging and dementia. With an annual progression rate of approximately 10%–15% from MCI to a formal AD diagnosis, there is an urgent need to identify individuals at highest risk.^1^^,^^2^ The development of robust, non-invasive biomarkers capable of accurately predicting this transition is therefore a key priority in AD research, as they can help identify individuals at higher risk of progression who are most likely to benefit from early intervention, support efficient design of clinical trials of emerging disease-modifying therapies, and inform patient counseling and day-to-day clinical management.^3^^,^^4^

However, MCI is not a monolithic clinical entity; it is characterized by significant heterogeneity in its clinical presentation, cognitive profiles, and underlying neuropathological substrates.^5^^,^^6^ Extensive research has demonstrated that different MCI subtypes, such as amnestic versus non-amnestic, exhibit distinct rates of progression and may evolve into different dementia syndromes.^7^ This clinical variability strongly suggests the existence of multiple, distinct neurodegenerative trajectories from MCI to AD, each potentially driven by unique patterns of brain pathology.^8^ A singular, one-size-fits-all approach to prognosis may therefore obscure crucial differences in disease mechanisms. Consequently, moving beyond a uniform view of MCI conversion and instead identifying these distinct prognostic subtypes is a crucial, yet underexplored, step toward a more precise understanding of AD pathogenesis and the development of targeted, personalized therapeutic strategies.

Structural magnetic resonance imaging (sMRI) is a widely accessible and non-invasive modality for quantifying brain atrophy, a core neuropathological feature of AD.^5^^,^^9^ To move beyond conventional volumetric analyses, radiomics enables the high-throughput extraction of a vast array of quantitative features that capture subtle, sub-visual information about tissue intensity, texture, and shape heterogeneity within specific brain regions.^10^ Complementing this intra-regional characterization, regional radiomics similarity networks (R2SNs) offer a powerful framework for mapping the brain’s macro-level organization. By constructing networks where nodes are brain regions and edges represent the similarity of their high-dimensional radiomic feature vectors, R2SNs provide a more comprehensive and sensitive measure of inter-regional morphological covariance than networks based on single anatomical markers.^11^^,^^12^ We hypothesize that the integration of local radiomic features with network-based R2SN metrics can provide a multi-scale neuroanatomical signature, uniquely positioned to capture the complex and spatially distributed patterns of brain alterations that may define the distinct pathways of MCI-to-AD progression.

**Figure 1:**
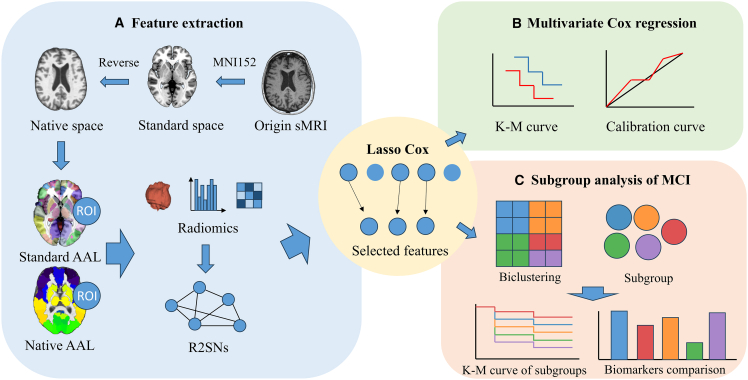
Schematic overview of the analytical pipeline (A) Feature extraction. sMRI were preprocessed and spatially registered to both MNI standard space and the participant’s native space. The AAL-116 atlas was applied in both spaces to define ROIs. From these ROIs, a comprehensive set of radiomic features was extracted, which were then used to construct R2SNs and compute network metrics. (B) Multivariate Cox regression. The combined feature set from both spaces underwent a LASSO-Cox feature selection process to identify a final prognostic signature (ARN-Sig). This signature was used to build a multivariate Cox proportional hazards model for predicting MCI-to-AD conversion, evaluated via K-M survival curves, C-index, and risk score distributions. (C) Subgroup analysis of MCI. The same ARN-Sig was used to perform a biclustering analysis, identifying distinct MCI subgroups. These subgroups were subsequently validated and characterized by comparing their survival distributions (K-M curves) and baseline biomarker profiles.

This study aimed to leverage this integrated imaging framework to improve prognostication and subtyping in MCI. We first extracted anatomical radiomic and network (R2SN) features from sMRI data. A feature-selection pipeline was then applied to identify a set of imaging factors associated with time to AD conversion, referred to as anatomical radiomic-network signature (ARN-Sig). This ARN-Sig was used for two main purposes: (1) to develop and validate a multivariate Cox regression model for predicting individual conversion risk and (2) to perform biclustering analyses to identify data-driven, prognostically distinct MCI subgroups. Finally, we characterized these subgroups in terms of their at-risk brain regions, survival patterns, and baseline clinical, cognitive, and biomarker profiles (Figure 1).

## Results

### Demographic characteristics

Demographic data for the ADNI (*n* = 274 pMCI, *n* = 224 sMCI) and NACC (*n* = 144 pMCI, *n* = 200 sMCI) cohorts are presented in Table 1. In both cohorts, pMCI participants were significantly older and had a higher prevalence of APOE ε4 carriers (all *p* < 0.05) compared to sMCI participants. Years of education did not differ significantly in either cohort (*p* > 0.05). Sex distribution differed significantly in the ADNI cohort with fewer females in the pMCI group (*p* < 0.05), but not in the NACC cohort (*p* = 0.27).

**Table 1:** Demographic characteristics of study participants

Datasets	Groups (*N*)	Age (years)	Sex (female, %)	Education (years)	APOE ε4 (carrier, %)
ADNI	pMCI (*n* = 274)	75 (71, 80)	41.24%	16 (14, 18)	49.64%
ADNI	sMCI (*n* = 224)	72 (67, 77)	61.16%	16 (14, 18)	30.80%
ADNI	*p* value	<0.05	<0.05	0.883	<0.05
NACC	pMCI (*n* = 144)	74 (68, 79)	54.17%	16 (15, 18)	40.03%
NACC	sMCI (*n* = 200)	72 (66, 77)	56.00%	16 (14, 18)	33.50%
NACC	*p* value	<0.05	0.270	0.783	<0.05

Age and education are presented as median (IQR). ADNI, Alzheimer’s Disease Neuroimaging Initiative; NACC, National Alzheimer’s Coordinating Center; pMCI, progressive mild cognitive impairment; sMCI, stable mild cognitive impairment.

### Feature selection results

Out of an initial total of 25,528 features (24,824 from radiomics and 704 from R2SNs), 24 features were retained following the feature selection process to constitute the ARN-Sig (Table 2). This final set included 16 features from native space and 8 from standard space, consisting of 19 radiomics features and 5 R2SNs features (local efficiency, degree, efficiency, and clustering). The ARN-Sig comprised features from 14 distinct brain regions, including amygdala, fusiform, hippocampus, precuneus, olfactory, rectus, and various frontal, parietal, occipital, and temporal lobe regions (frontal superior, frontal superior orbital, occipital middle, parietal inferior, temporal inferior, temporal middle, temporal pole middle, and parahippocampal).

**Table 2:** Performance of the prognostic models in the NACC validation cohort

Metric	Model 1	Model 2	Model 3
C-index	0.76	0.73	0.71
AUC (*t* = 1 year) (95% CI)	0.78 (0.70–0.85)	0.73 (0.65–0.81)	0.70 (0.61–0.78)
AUC (*t* = 2 years) (95% CI)	0.81 (0.76–0.86)	0.76 (0.71–0.82)	0.74 (0.68–0.80)
AUC (*t* = 3 years) (95% CI)	0.82 (0.77–0.87)	0.79 (0.74–0.84)	0.78 (0.73–0.82)
ΔNB (*t* = 1 year)	0.02	0	0
ΔNB (*t* = 2 years)	0.12	0.09	0.08
ΔNB (*t* = 3 years)	0.11	0.10	0.09

Model 1, dual-space ARN-Sig model; model 2, conventional multi-region volumetric MRI model; model 3, clinical model. ΔNB, change in net benefit.

### Result of multivariate Cox model

**Figure 2:**
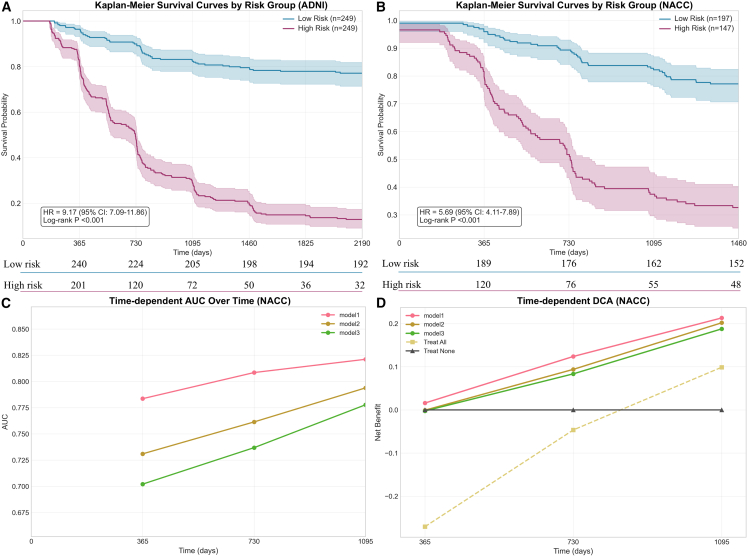
Prognostic stratification, discrimination, and decision-curve performance of the ARN-Sig model and comparator models (A and B) K-M survival curves for progression from MCI to AD stratified by the ARN-Sig model in the (A) ADNI training cohort and (B) NACC external validation cohort. In both cohorts, participants were divided into high-risk (red) and low-risk (blue) groups using the median risk score derived from the ADNI training set. Shaded areas indicate 95% confidence intervals. (C) Time-dependent AUCs of the three prognostic models in the NACC cohort at 1, 2, and 3 years. Model 1 denotes the ARN-Sig model; model 2, the conventional multi-region structural MRI volumetric model; and model 3, the clinical-only model. (D) Time-dependent decision curve analysis (DCA) of the three models in the NACC cohort. Model 1 showed the highest net benefit across the evaluated time points compared with model 2, model 3, and the treat-all and treat-none strategies. See also Figures S1 and S2 and Tables S1–S4.

Compared with the two baseline Cox models, model 1 (ARN-Sig) demonstrated the best prognostic performance in the external NACC cohort (Table 2; Figures 2C and 2D). The C-index of model 1 was 0.76, which was higher than that of model 2 (0.73) and model 3 (0.71). Similarly, the time-dependent AUCs of model 1 at 1, 2, and 3 years were 0.78 (95% CI: 0.70–0.85), 0.81 (95% CI: 0.76–0.86), and 0.82 (95% CI: 0.77–0.87), respectively, consistently exceeding the corresponding values of model 2 [0.73 (0.65–0.81), 0.76 (0.71–0.82), and 0.79 (0.74–0.84)] and model 3 [0.70 (0.61–0.78), 0.74 (0.68–0.80), and 0.78 (0.73–0.82)]. Pairwise comparisons further confirmed that the 1-, 2-, and 3-year AUCs of model 1 were each significantly higher than the corresponding AUCs of model 2 and model 3 (all *p* < 0.05). DCA further showed that model 1 provided the greatest clinical utility across all evaluated time points, with ΔNB values of 0.02, 0.12, and 0.11 at 1, 2, and 3 years, respectively, compared with 0, 0.09, and 0.10 for model 2 and 0, 0.08, and 0.09 for model 3. The results in the ADNI validation cohort are presented in Table S1. Calibration curves, as well as the corresponding slope and intercept values, are presented in Figures S1 and S2 and Tables S2 and S3.

KM survival analyses based on the predefined risk-score cutoff showed that model 1 achieved robust risk stratification in both cohorts (Figures 2A and 2B). In the ADNI cohort, model 1 separated the high-risk and low-risk groups with a hazard ratio (HR) of 9.17 (95% CI: 7.09–11.86; log rank *p* < 0.001). This prognostic separation remained significant in the external NACC cohort, with an HR of 5.69 (95% CI: 4.11–7.89; log rank *p* < 0.001). Consistent with the survival results, the time-dependent AUC curves also showed that model 1 maintained superior discriminative ability over model 2 and model 3 throughout follow-up. For all Cox regression models, Schoenfeld residual tests indicated no significant violation of the proportional hazards assumption (all *p* > 0.05).

Ablation analyses further demonstrated that ARN-Sig achieved a higher C-index (0.80) than the standard-space-only model (0.77), native-space-only model (0.74), radiomics-only model (0.76), and R2SN-only model (0.75). Likewise, the time-dependent AUCs of ARN-Sig at each evaluated time point were consistently higher than those of all four single-component models. Detailed results of the ablation analyses are provided in Table S4.

### Identification of MCI subtypes based on ARN-Sig

#### Biclustering identification of MCI subtypes

**Figure 3:**
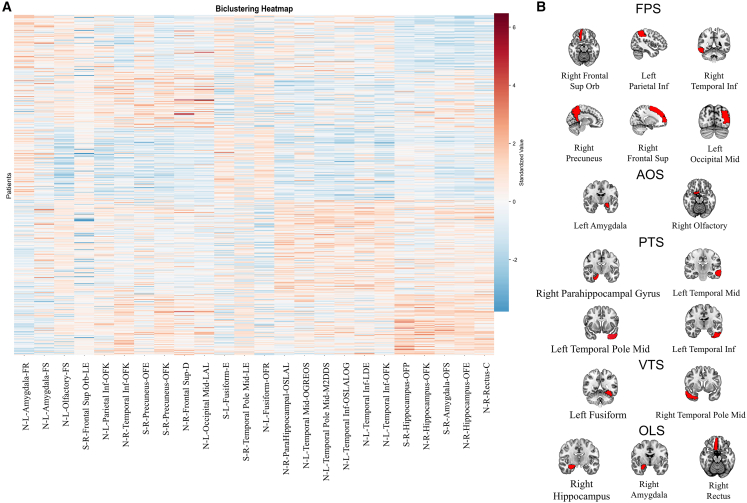
Biclustering analysis of the ARN-Sig for MCI subtype identification (A) Biclustering heatmap of the standardized ARN-Sig feature matrix. The analysis simultaneously clusters patients (rows) and features (columns), revealing distinct patient subgroups and their associated feature patterns. Colors represent the standardized *Z* score of each feature, from low (blue) to high (red). Feature names on the *x* axis are prefixed as follows: “L” for left hemisphere, “R” for right hemisphere, “N” for native space, and “S” for standard space. (B) Anatomical visualization of the five data-driven MCI subtypes identified by the biclustering analysis. The brain regions (highlighted in red) correspond to the key features defining each prognostic pathway: FPS, AOS, PTS, VTS, and OLS. See also Tables S5 and S6.

We applied biclustering to the ARN-Sig data matrix across all patients to identify subtypes with shared feature profiles. Among the tested cluster solutions, the five-cluster solution (*k* = 5) achieved the highest overall clustering score and revealed five distinct patient subtypes, each defined by a unique set of features mapping to specific anatomical pathways (Figures 3A and 3B; Table 2). Detailed clustering scores for *k* = 3 to *k* = 6 are provided in Table S5. These five subtypes, each defined by distinct neuroanatomical features: (1) a fronto-parietal subtype (FPS) involving superior frontal, orbital, inferior parietal, and middle occipital regions; (2) an amygdala-olfactory subtype (AOS); (3) a parahippocampal-temporal subtype (PTS) encompassing the parahippocampal gyrus and temporal pole; (4) a ventral temporal subtype (VTS) involving the fusiform gyrus and temporal pole; and (5) an orbitofrontal-limbic subtype (OLS) centered on the hippocampus, amygdala, and rectus gyrus. A comprehensive list of the specific features defining each pathway is provided in Table S6.

#### KM survival analysis of MCI subtypes

KM survival curves were used to compare the different risks of progression from MCI to AD among the five identified subtypes. As shown in Table 3, the overall log rank test confirmed a highly significant difference in survival distributions among the subtypes in both the ADNI training set (*p* < 0.001) and the NACC external validation set (*p* < 0.001).

**Table 3:** Survival analysis based on MCI subgroups in ADNI and NACC cohorts

Cohorts	Subgroups	Events/total (*N*)	Grouping^1^
ADNI	overall log rank test	–	*p* < 0.001
ADNI	FPS	70/80	a
ADNI	AOS	50/85	b
ADNI	PTS	90/106	a
ADNI	VTS	50/138	c
ADNI	OLS	14/89	d
NACC	overall log rank test	–	*p* < 0.001
NACC	FPS	29/42	a
NACC	AOS	17/36	b
NACC	PTS	57/91	a, b
NACC	VTS	28/96	c
NACC	OLS	13/79	d

Grouping^1^ based on post hoc pairwise log-rank tests (*p* < 0.05, adjusted for FDR). Groups sharing the same letter are not statistically different. FPS, fronto-parietal subtype; AOS, amygdala-olfactory subtype; PTS, parahippocampal-temporal subtype; VTS, ventral temporal subtype; OLS, orbitofrontal-limbic subtype.

**Figure 4:**
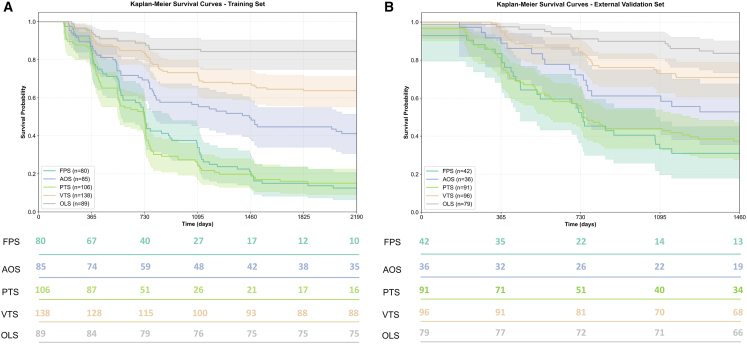
Prognostic significance of the five identified MCI subtypes K-M survival curves showing AD-conversion–free survival (probability of remaining free from progression from MCI to AD) for the five identified subtypes: Fronto-Parietal Subtype (FPS), Amygdala-Olfactory Subtype (AOS), Parahippocampal-Temporal Subtype (PTS), Ventral Temporal Subtype (VTS), and Orbitofrontal-Limbic Subtype (OLS). The survival distributions are presented for (A) the ADNI training set and (B) the NACC external validation set. The tables below each plot indicate the number of patients at risk at different time points for each subtype. See also Tables S7 and S8.

In the ADNI cohort, KM curves showed clear stratification among subtypes (Figure 4A). The FPS and PTS subtypes exhibited the poorest prognosis, with the most rapid progression to conversion. *Post hoc* pairwise log rank tests confirmed that FPS and PTS carried a similarly high risk, significantly greater than all others. The AOS, VTS, and OLS subtypes each represented statistically distinct risk tiers—intermediate, low, and very-low, respectively.

This prognostic stratification was successfully replicated in the external NACC validation cohort (Figure 4B). A consistent risk pattern emerged: the FPS and PTS subtypes again formed a statistically indistinguishable high-risk group, while the VTS and OLS subtypes consistently demonstrated the lowest risk and were statistically distinct from all others. Notably, the AOS subtype exhibited an intermediate risk level that was not statistically different from the PTS subtype (Table 3). Detailed absolute conversion rates for each subtype at each time point are provided in Tables S7 and S8.

#### Baseline characteristics of MCI subtypes

**Figure 5:**
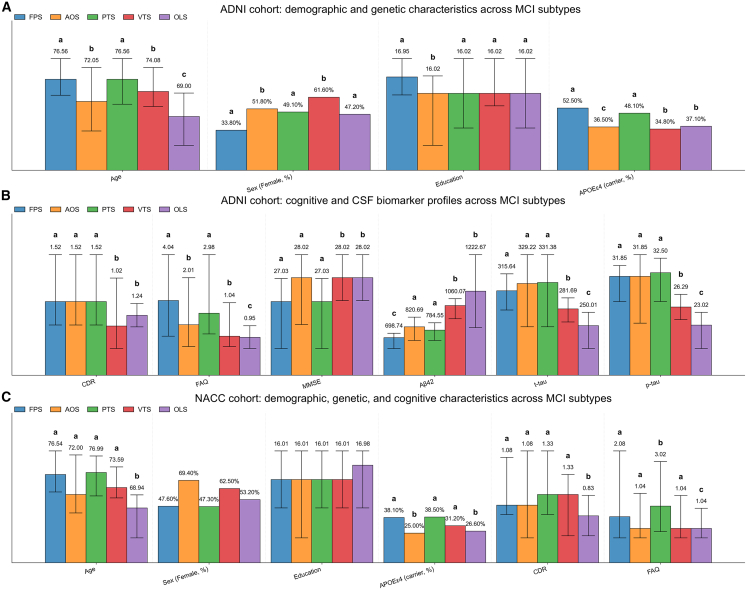
Baseline demographic, clinical, and biomarker characteristics of the five MCI subtypes (A) Demographic and genetic characteristics in the ADNI cohort. (B) Cognitive and CSF biomarker profiles in the ADNI cohort. (C) Demographic, genetic, and cognitive characteristics in the NACC cohort. Bars represent median values with interquartile ranges for continuous variables and percentages for categorical variables. Lowercase letters above the bars indicate compact-letter groupings derived from post hoc pairwise comparisons performed separately for each variable. For a given variable, subtypes sharing at least one letter are not significantly different, whereas subtypes with no shared letters are significantly different after FDR correction (adjusted *p* < 0.05). The letters are specific to each variable and do not imply an ordinal ranking. Detailed pairwise comparison results are provided in Data S1. FPS, fronto-parietal subtype; AOS, amygdala-olfactory subtype; PTS, parahippocampal-temporal subtype; VTS, ventral temporal subtype; OLS, orbitofrontal-limbic subtype.

Baseline demographic characteristics were consistent across cohorts. In both ADNI and NACC, individuals in the high-risk FPS and PTS subtypes were significantly older than those in the lowest-risk OLS subtype (Figures 5A and 5C). Sex distribution also varied consistently by subtype. The AOS subtype (and VTS in NACC) had a higher proportion of females, whereas the FPS subtype had the lowest (Figures 5A and 5C). In contrast, years of education did not differ significantly among the five subtypes in either cohort (Figures 5A and 5C).

Clinical measures further differentiated the subtypes. In the ADNI cohort, FPS and PTS demonstrated greater functional impairment (FAQ) and higher clinical severity (CDR-SB) compared with the low-risk OLS group (Figure 5B). These differences were replicated in the NACC cohort, where FAQ differed across subtypes and CDR was significantly lower in OLS than in the other subtypes (Figure 5C). Cognitive performance showed a similar trend, with FPS and PTS exhibiting significantly lower MMSE scores than OLS in ADNI (Figure 5B). Genetic susceptibility aligned with the clinical profile; FPS and PTS had a higher prevalence of APOE ε4 carriers than AOS and OLS in ADNI (Figure 5A), and this pattern was also observed in NACC, where FPS and PTS showed higher APOE ε4 carrier prevalence than AOS and OLS (Figure 5C).

CSF analyses in ADNI revealed that the FPS and PTS subtypes had substantially higher levels of p-tau and t-tau compared with the remaining subtypes (Figure 5B). A gradient was also evident in CSF Aβ42, with FPS showing the lowest levels, consistent with more advanced amyloid pathology and significantly lower than the OLS subtype (Figure 5B). Together, these biomarker profiles support the distinction between the high-risk (FPS, PTS) and low-risk (VTS, OLS) subtypes identified in both cohorts.

## Discussion

In this study, we developed and externally validated a dual-space sMRI-based anatomical radiomic-network signature (ARN-Sig) to predict MCI-to-AD conversion and to characterize heterogeneity within MCI. In both the ADNI training cohort and the NACC validation cohort, Cox models based on the ARN-Sig demonstrated good discriminative performance and consistent risk stratification, with higher C-indices and clearer separation of progression curves than the regional volumetric model and the clinical-only model. Using the same ARN-Sig feature set, we further identified five MCI subtypes—FPS, PTS, AOS, VTS, and OLS—that displayed distinct conversion risks, structural patterns, and baseline clinical, genetic, and CSF profiles across both datasets.

A key finding of this study is that the five ARN-Sig-derived MCI subtypes are not only structurally distinct, but also map onto systematically different clinical, genetic, and CSF profiles. Among them, the FPS and PTS subtypes form a convergent high-risk axis. The FPS cluster is defined by structural alterations in precuneus, inferior parietal, and superior frontal regions, while the PTS subtype predominantly involves the parahippocampal gyrus and anterior temporal areas. These regions are core hubs of the episodic memory and default mode networks, systems particularly vulnerable in early AD and tightly linked to rapid clinical decline.^13^^,^^14^ In our cohorts, patients assigned to FPS and PTS consistently showed the highest functional impairment (FAQ, CDR-SB), the lowest global cognitive performance (MMSE), the greatest enrichment of APOE ε4 carriers, and the most AD-consistent CSF pattern, with markedly increased p-tau and t-tau and reduced Aβ42 compared with the remaining subtypes. The alignment between these structurally defined pathways and a more advanced clinical and biomarker profile across two independent cohorts supports the interpretation that FPS and PTS capture MCI individuals who are already positioned on an aggressive, AD-like progression trajectory.^15^^,^^16^^,^^17^

By contrast, the remaining three subtypes illustrate that a substantial proportion of MCI follows structurally and biologically milder trajectories. The OLS subtype, characterized by orbitofrontal and limbic involvement including the hippocampus, amygdala, and rectus gyrus, showed the lowest conversion rates together with relatively preserved cognition and the most favorable CSF profile, with lower tau levels and higher Aβ42. This combination suggests that, in these patients, limbic-predominant structural change may represent a more restricted and biologically milder expression of AD-related pathology, with relatively limited extension into broader association neocortical networks, which is consistent with their slower clinical deterioration.^18^^,^^19^^,^^20^ The VTS subtype, defined by fusiform and ventral temporal features, also exhibited low progression risk. Given the prominent role of the ventral temporal stream in higher-order visual processing, structural alterations that are relatively confined to this pathway may have a less direct impact on the core episodic memory and default mode-related systems that drive rapid AD progression, which is consistent with the comparatively preserved baseline cognition and intermediate biomarker changes observed in this group.^21^^,^^22^ The AOS subtype, anchored in amygdala and olfactory regions, occupied an intermediate position: its conversion risk, cognitive performance, and CSF measures consistently fell between the high-risk (FPS, PTS) and low-risk (OLS, VTS) subtypes. This pattern is compatible with a trajectory in which early involvement of olfactory-limbic structures marks an at-risk state, but widespread neocortical engagement has not yet occurred.^23^^,^^24^ Taken together, these three subtypes underscore that not all MCI with structural abnormalities is biologically equivalent. Distinct constellations of regional atrophy, APOE ε4 burden, and CSF alterations were associated with clearly different risks and may reflect multiple underlying disease mechanisms within the MCI spectrum. At the same time, we cannot exclude the possibility that part of the observed subtype separation reflects differences in baseline disease burden or stage, rather than fully distinct biological pathways. These subtypes should therefore be interpreted conservatively as imaging-defined prognostic phenotypes that capture both anatomical heterogeneity and disease severity.

The performance advantage of ARN-Sig suggests that the complexity of MCI-to-AD progression is not fully captured by conventional clinical variables or a limited set of regional brain volume measures.^25^^,^^26^ Clinical factors and regional atrophy markers provide clinically meaningful and interpretable baselines, but their weaker prognostic performance in the external validation cohort indicates that downstream risk is shaped by more distributed and heterogeneous structural alterations. In contrast, ARN-Sig integrates high-throughput radiomic features with R2SN-derived network metrics, thereby capturing both local tissue characteristics (intensity, texture, and shape) and inter-regional morphological covariance across multiple brain regions.^27^^,^^28^^,^^29^^,^^30^ This multi-scale representation appears better suited to summarize the distributed neuroanatomical patterns associated with conversion risk. Consistent with this interpretation, the ablation results further support the incremental value of combining radiomic and network information within a unified framework. While further work is needed to refine and test this approach in broader settings, our results suggest that moving beyond clinical variables or regional volumetry toward integrated anatomical radiomic-network signatures may provide more informative prognostic markers for MCI.

The present findings have several practical implications for both clinical care and trial design in MCI. Because the ARN-Sig is derived from routine sMRI, it could, after prospective validation, be implemented in memory clinics as an automated risk score that complements standard clinical assessment.^31^ In particular, patients assigned to high-risk subtypes such as FPS and PTS—who consistently show the highest conversion rates and the most AD-consistent biomarker profiles—would be readily identifiable as candidates for closer follow-up, earlier biomarker work-up, and preferential enrollment into disease-modifying trials, where enrichment for rapid progressors is needed to increase statistical power.^32^^,^^33^^,^^34^ Overall, our stratification framework is best viewed as a complementary tool for constructing more homogeneous trial cohorts and generating more precise hypotheses about differential disease trajectories within the MCI spectrum, rather than as the sole determinant of individual treatment decisions. Consistent with the DCA findings, this suggests that ARN-Sig may offer complementary clinical utility as a decision-support tool, although prospective studies are still needed to determine whether its use translates into measurable improvements in real-world clinical decision-making.

In conclusion, this study develops and externally validates a dual-space sMRI-based ARN-Sig that improves prediction of MCI-to-AD conversion and delineates five structurally and biologically distinct MCI subtypes. These findings support ARN-Sig-guided stratification as a potentially useful framework for refining individual risk assessment and designing more homogeneous cohorts in future clinical trials.

### Limitations of the study

Several limitations of this study warrant consideration. First, although the ARN-Sig and the five subtypes were validated in two large, independent multicenter cohorts (ADNI and NACC), participants were predominantly highly educated, white research volunteers. Replication in larger, more diverse, population-based clinical samples will be important to establish the generalizability and clinical utility of our findings across different ethnicities and socioeconomic backgrounds. Second, we deliberately focused on sMRI-based radiomic and morphological covariance measures because of their wide availability, non-invasive nature, and relatively low cost. This focus enhances the potential for clinical translation but also means that our models do not incorporate microstructural or functional imaging information, which may capture complementary aspects of disease biology. Future work could therefore examine whether combining the ARN-Sig with diffusion- or functional-imaging-derived metrics provides incremental prognostic value beyond the current sMRI framework, particularly in settings where such multimodal data are routinely acquired.

## Resource availability

### Lead contact

Further information and requests for resources should be directed to and will be fulfilled by the lead contact, Junping Wang (wangjunping_tj@163.com).

### Materials availability

This study did not generate new unique reagents, biological materials, or other physical materials.

### Data and code availability

This paper analyzes baseline T1-weighted structural MRI, demographic, clinical, genetic, cognitive, and biomarker data obtained from the Alzheimer’s Disease Neuroimaging Initiative (ADNI) and the National Alzheimer’s Coordinating Center (NACC). ADNI and NACC data are available to qualified researchers upon application to the respective data repositories and are subject to their data-use agreements and governance requirements. The analysis code used in this study is publicly available at https://github.com/bearsyep/iscience_R2SN_radiomics. Additional information required to reanalyze the data reported in this paper is available from the lead contact upon reasonable request, subject to the restrictions of the source cohorts.

## Acknowledgments

This work was funded by 10.13039/501100010590Tianjin Health Research Project (TJWJ2025ZD001) and Tianjin Science and Technology Plan Project (25ZXWZSY00070).

## Author contributions

D.X., M.L., and Z.L. jointly conceptualized and designed the study; D.X. and Z.L. performed data preprocessing, statistical analysis, and visualization; J.X. contributed to data collection, data preprocessing, and manuscript review; D.X. drafted the initial manuscript; M.L., Z.L., and J.X. contributed to the interpretation of the results and critically reviewed and revised the manuscript for important intellectual content; J.W. supervised the study, provided methodological guidance, critically revised the manuscript, and obtained funding. All authors approved the final version of the manuscript and agree to be accountable for all aspects of the work.

## Declaration of interests

The authors declare that they have no competing interests.

## STAR★Methods

### Key resources table

**Table:** 

REAGENT or RESOURCE	SOURCE	IDENTIFIER
**Deposited data**		

Alzheimer’s Disease Neuroimaging Initiative baseline T1-weighted MRI and associated clinical data	Alzheimer’s Disease Neuroimaging Initiative (ADNI)	https://adni.loni.usc.edu/; access upon application
National Alzheimer’s Coordinating Center baseline T1-weighted MRI and associated clinical data	National Alzheimer’s Coordinating Center (NACC)	https://naccdata.org/; access upon application
Analysis code for the dual-space anatomical radiomic-network signature workflow	This paper	https://github.com/bearsyep/iscience_R2SN_radiomics

**Software and algorithms**		

Python	Python Software Foundation	Version 3.10; https://www.python.org/
PyRadiomics	PyRadiomics project	Version 3.0.1; https://pyradiomics.readthedocs.io/
scipy.stats	SciPy project	Version 1.10.1; https://scipy.org/
lifelines	lifelines project	Version 0.27.7; https://lifelines.readthedocs.io/
SPM12	Wellcome Center for Human Neuroimaging	https://www.fil.ion.ucl.ac.uk/spm/software/spm12/
Computational Anatomy Toolbox for SPM12	CAT12 project	Version 12.8; https://neuro-jena.github.io/cat/
DARTEL spatial normalization algorithm	SPM12	Implemented in SPM12

**Other**		

Automated Anatomical Labeling atlas, 116 regions	AAL atlas	AAL-1; https://www.gin.cnrs.fr/en/tools/aal/

### Experimental model and study participant details

#### Human participants and cohorts

Baseline T1-weighted structural MRI data were obtained from two human MCI cohorts: ADNI and NACC. The ADNI cohort served as the derivation cohort and included 498 individuals with MCI, comprising 274 progressive MCI (pMCI) and 224 stable MCI (sMCI) cases, with follow-up of up to 2,190 days (6 years). The NACC cohort served as an independent external validation cohort and included 344 individuals with MCI, comprising 144 pMCI and 200 sMCI cases, with follow-up of up to 1,460 days (4 years). Definitions of MCI, pMCI, and sMCI are provided in Method S1.

Participant age, sex, years of education, APOE ε4 carrier status, baseline cognitive assessments, and cerebrospinal fluid (CSF) biomarkers were extracted when available. Sex was evaluated in baseline comparisons and included as a covariate in the multivariable Cox models. Harmonized ethnicity/race information was not included in the present cross-cohort analyses because the modeling framework used variables available across both cohorts. No sex- or gender-stratified modeling was performed.

#### Ethics statement

The ADNI and NACC studies were conducted in accordance with the Declaration of Helsinki. For ADNI, the study protocols were approved by the institutional review boards or research ethics boards of the participating institutions, and written informed consent was obtained from all participants or their legally authorized representatives. For NACC, written informed consent was obtained from participants at the contributing Alzheimer’s Disease Centers, with approval from each center’s local institutional review board; research using the NACC database was approved by the University of Washington Institutional Review Board (IRB No. 36178). The present study used de-identified data from these existing cohorts and did not involve new participant recruitment, new sample collection, or experimental intervention. This study was not an interventional clinical trial, and no clinical trial registration applies.

### Method details

#### MRI preprocessing and spatial registration

A standardized preprocessing pipeline was applied to all T1-weighted MRI scans. Initial preprocessing included image denoising and N4 bias field correction to reduce intensity non-uniformity. The processed images were spatially normalized to Montreal Neurological Institute (MNI) standard space using the Diffeomorphic Anatomical Registration Through Exponentiated Lie Algebra (DARTEL) algorithm, and the resulting images were resampled to an isotropic voxel size of 1 × 1 × 1 mm^3^. Voxel intensities were then normalized using a *Z* score transformation.

The Automated Anatomical Labeling (AAL) atlas was warped onto each MNI-normalized image to define 116 regions of interest (ROIs). To generate native-space data, the inverse DARTEL deformation fields were applied to the MNI-normalized T1-weighted images and the AAL atlas, producing an individualized atlas and a T1-weighted image in each participant’s native space. Native-space images also underwent *Z* score intensity normalization, and the same 116 AAL regions were used as ROIs.

Both MNI standard space and participant-specific native space were analyzed because they provide complementary information. Standard space supports consistent atlas-based parcellation and anatomical correspondence across participants, whereas native space preserves participant-specific morphology and local contrast after registration, potentially capturing additional inter-individual variability relevant to radiomic and network-based features.

#### Radiomic feature extraction

Radiomic features were extracted from the 116 AAL-defined ROIs in both MNI standard space and native space images using the PyRadiomics library. For each ROI, 107 radiomic features were computed, including 18 first-order statistical features, 14 shape-based features, and 75 texture features. Texture features were derived from five matrices: the gray level co-occurrence matrix (GLCM; 24 features), gray level run length matrix (GLRLM; 16 features), gray level size zone matrix (GLSZM; 16 features), gray level dependence matrix (GLDM; 14 features), and neighboring gray tone difference matrix (NGTDM; 5 features). This procedure generated two regional radiomic feature sets per participant, one from MNI standard space and one from native space, each containing 12,412 features (107 features × 116 ROIs).

#### Regional radiomics similarity network construction

Two regional radiomics similarity networks (R2SNs) were constructed for each participant, one in MNI standard space and one in native space. In each network, the 116 AAL regions were treated as nodes. Edge weights were defined as Pearson correlations between the 107-dimensional radiomic feature vectors of each pair of regions after feature scaling and removal of inter-correlations greater than 0.90. The resulting 116 × 116 matrices were binarized across a threshold range from 0.50 to 0.75 with a step size of 0.01.

Graph-theoretic metrics were computed from the binarized graphs and averaged across thresholds. This generated two network feature sets per participant, one from MNI standard space and one from native space. Each network feature set contained 352 features, comprising 4 global metrics and 3 nodal metrics measured across 116 ROIs.

#### Regional brain volume extraction

For the regional volumetric comparator model, MNI-space T1-weighted images were segmented into gray matter (GM), white matter, and CSF probability maps using the Computational Anatomy Toolbox for SPM12. This procedure generated modulated GM segments in which voxel intensities were adjusted to compensate for volume changes introduced during DARTEL normalization, thereby preserving original tissue volume information in standard space. Using the AAL atlas warped to MNI space, masks were generated for all predefined brain regions. The GM volume of each ROI was calculated by summing voxel values within the corresponding regional mask from the modulated GM segment images.

#### Feature preprocessing and ARN-Sig derivation

Within the ADNI derivation cohort, all feature selection steps were performed within a cross-validation framework to avoid information leakage. For each training fold, features first underwent variance filtering with a threshold of 0.1. Retained features then underwent correlation-based redundancy reduction using Spearman correlation distance. Within each correlation cluster, only the feature showing the strongest association with time to AD conversion in univariate Cox analysis was retained. These procedures were applied using the training partition only and were repeated independently in each fold.

A LASSO-Cox model with 5-fold cross-validation was then fitted within the training data to select the optimal penalty parameter and identify features with non-zero coefficients. The trained model was applied to the corresponding held-out fold to generate risk scores and estimate internal performance. After completing cross-validation, the final anatomical radiomic-network signature (ARN-Sig) was derived by reapplying the same feature-selection pipeline to the full ADNI derivation cohort and was subsequently evaluated in the independent NACC cohort.

#### Cox model development and validation

A multivariable Cox proportional hazards model (model 1) was constructed using the ARN-Sig features and adjusted for age, sex, years of education, APOE ε4 carrier status, and baseline cognitive assessment. These covariates were selected *a priori* based on prior AD/MCI prognostic studies.^35^^,^^36^^,^^37^ Two comparator models were constructed using the same clinical covariates: model 2, a regional volumetric model based on volumetric measures from all brain regions plus the clinical covariates; and model 3, a clinical-only model including age, sex, education, APOE ε4 status, and baseline cognitive assessment.

For model 1 and model 2, feature preprocessing and prognostic modeling followed the same pipeline, including variance filtering, correlation-based redundancy reduction, univariate Cox screening, and 5-fold cross-validated LASSO-Cox feature selection in the ADNI training cohort. Selected imaging features were entered into multivariable Cox proportional hazards models together with the clinical covariates and evaluated in both ADNI and NACC.

To evaluate risk stratification, the median risk score derived from the ADNI training cohort was used as the cutoff to assign participants in both the training cohort and the external validation cohort to high-risk and low-risk groups. Kaplan-Meier survival analyses were then performed. Tail-end time points were excluded from these analyses (2,190 days in ADNI and 1,460 days in NACC) because follow-up was insufficient for many participants at these late times, resulting in a small number of individuals remaining at risk and unstable survival estimates.

#### Ablation analysis

To assess the incremental contribution of spatial domains and feature types, ablation analyses were performed using the same training-validation framework as the main prognostic model. Five candidate models were compared: (1) a standard-space-only model including radiomic and R2SN features extracted only from MNI standard space; (2) a native-space-only model including radiomic and R2SN features extracted only from participant-specific native space; (3) a radiomics-only model including radiomic features from both spaces but excluding R2SN metrics; (4) an R2SN-only model including network metrics from both spaces but excluding regional radiomic features; and (5) the combined ARN-Sig model integrating radiomic and R2SN features from both spaces. All ablation models used the same feature-selection strategy, model construction procedure, and performance evaluation criteria as the main prognostic model.

#### Biclustering-based MCI subtype identification and validation

To identify imaging-defined MCI subtypes, biclustering analysis based on ARN-Sig was performed in the ADNI cohort. After *Z* score standardization of the ARN-Sig feature matrix, the Spectral Co-Clustering algorithm was applied to simultaneously cluster patients (rows) and features (columns). Candidate solutions with k = 3 to k = 6 clusters were compared.

Clustering quality was evaluated using three metrics: silhouette score, which assessed cluster compactness and separation; log rank test statistic, which assessed survival discrimination among clusters; and block R^2^, which assessed bicluster structural coherence. These metrics were normalized across candidate k values and averaged with equal weights to generate a composite score. The log rank statistic was transformed so that higher values indicated better performance. The solution with the highest composite score was selected as optimal.

Cluster centroids derived from the standardized ADNI training data were used to assign NACC validation participants to the nearest cluster according to minimum Euclidean distance after standardizing the validation data using the training-set parameters. The prognostic significance of the resulting clusters was assessed in both cohorts, with survival times right-censored at 2,160 days in ADNI and 1,460 days in NACC. Kaplan-Meier survival curves were generated for each subtype.

Baseline heterogeneity across MCI subtypes was assessed using available variables in each cohort. In ADNI, these variables included age, sex, years of education, APOE ε4 status, cognitive scores (Clinical Dementia Rating [CDR], Functional Activities Questionnaire [FAQ], and Mini-Mental State Examination [MMSE]), and CSF biomarkers (amyloid-beta 42 [Aβ42], total tau [t-tau], and phosphorylated tau [p-tau]). In NACC, the available corresponding variables included age, sex, years of education, APOE ε4 status, CDR, and FAQ.

### Quantification and statistical analysis

All statistical analyses were performed using Python version 3.10 with the scipy.stats and lifelines libraries. Continuous variables were assessed for normality using the Shapiro-Wilk test and were found to follow non-normal distributions. Therefore, non-parametric tests were used for group comparisons. Continuous variables are reported as median and interquartile range (IQR), and categorical variables are reported as counts and percentages.

For comparisons of continuous variables between two independent groups, the Mann-Whitney U test was used. For comparisons across three or more independent groups, including baseline characteristics among biclusters, the Kruskal-Wallis H test was used, followed by Dunn’s post-hoc pairwise test when a significant overall difference was detected. Differences in categorical variables, including sex and APOE ε4 carrier status, were assessed using the chi-squared test.

Cox model discrimination was evaluated using Harrell’s concordance index (C-index) and time-dependent area under the receiver operating characteristic curve (AUC). Calibration curves were used to assess model calibration, and decision curve analysis was used to evaluate clinical utility. In decision curve analysis, the change in net benefit (ΔNB) was defined as the difference in net benefit between each Cox model and the treat-all or treat-none strategy. Survival differences among the identified MCI subtypes were assessed using the multivariate log rank test, followed by pairwise log rank tests for post-hoc comparisons. The proportional hazards assumption of each Cox model was evaluated using Schoenfeld residuals. All *p* values were adjusted for multiple comparisons using the false discovery rate (FDR), with an adjusted *p* value less than 0.05 considered statistically significant.

## Supplemental information

Document S1. Figures S1 and S2, Tables S1–S8, and Methods S1

Data S1. Detailed statistical information for figure panels, related to Figures 5
