# Supplementary material for: A dual space MRI radiomic network signature for risk stratification and subtyping of mild cognitive impairment
Source: iScience. 2026 Jun 12;29(7):116394. doi: 10.1016/j.isci.2026.116394 (PMC13276304; doi:10.1016/j.isci.2026.116394)
Supplement: Document S1. Figures S1 and S2, Tables S1–S8, and Methods S1 [file mmc1.pdf]

iScience, Volume *29*

Supplemental information

A dual space MRI radiomic network signature for risk stratification and subtyping of mild cognitive impairment

Diaohan Xiong, Mengjiao Liu, Jiamei Xie, Zefeng Liu, and Junping Wang

Methods S1. Definition of MCI, sMCI, and pMCI

Mild cognitive impairment (MCI) was defined as the presence of measurable cognitive impairment without meeting criteria for dementia. Participants were required to have a Clinical Dementia Rating (CDR) global score of 0.5 with a memory box score ≥0.5, a Mini-Mental State Examination (MMSE) score of 24–30, and objective memory impairment on Logical Memory II (Delayed Recall) according to education-adjusted cutoffs: ≤8 for individuals with ≥16 years of education, ≤4 for those with 8–15 years of education, and ≤2 for those with 0–7 years of education. Amyloid-β (Aβ) positivity was defined using established biomarker thresholds, namely a global cortical Positron Emission Tomography standardized uptake value ratio (PET SUVR) ≥1.11 (whole cerebellum reference) or a cerebrospinal fluid (CSF) Aβ42/Aβ40 ratio <0.068.

Alzheimer's disease (AD) was defined as dementia consistent with AD, including a CDR global score of 0.5–1.0, an MMSE score of 20–26, and evidence of objective memory impairment based on the same education-adjusted Logical Memory II thresholds used for the MCI group. Aβ positivity in AD was defined using the same criteria as for MCI.

Based on longitudinal follow-up, participants with MCI were further categorized as stable MCI (sMCI) or progressive MCI (pMCI). Specifically, pMCI referred to individuals who met criteria for MCI at baseline but progressed to AD by the end of follow-up, whereas sMCI referred to individuals who remained diagnosed with MCI throughout the follow-up period without conversion to AD. In this study, the ADNI cohort served as the training dataset and included 498 participants with MCI, of whom

274 were classified as pMCI and 224 as sMCI, with follow-up of up to 2,190 days (6 years). The NACC cohort served as an independent external validation dataset and included 344 participants with MCI, comprising 144 pMCI and 200 sMCI cases, with a maximum follow-up of 1,460 days (4 years).

Table S1. Comparative performance of different models in the ADNI validation cohort

Metric	Model 1	Model 2	Model 3
C-index	0.80	0.74	0.72
AUC (t=1 year) (95% CI)	0.79 (0.75-0.86)	0.72 (0.66-0.80)	0.70 (0.65-0.78)
AUC (t=2 year) (95% CI)	0.85 (0.82-0.88)	0.78 (0.76-0.83)	0.76 (0.74-0.82)
AUC (t=3 year) (95% CI)	0.87 (0.85-0.90)	0.81 (0.79-0.86)	0.79 (0.77-0.84)
AUC (t=4 year) (95% CI)	0.89 (0.87-0.92)	0.82 (0.80-0.87)	0.80 (0.78-0.85)
AUC (t=5 year) (95% CI)	0.90 (0.89-0.93)	0.84 (0.82-0.89)	0.82 (0.80-0.87)
Δ NB (t=1 year)	0.02	0.01	0
Δ NB (t=2 year)	0.16	0.12	0.11
Δ NB (t=3 year)	0.10	0.08	0.08
Δ NB (t=4 year)	0.10	0.07	0.05
Δ NB (t=5 year)	0.10	0.06	0.05

Table S2. Calibration intercept and slope in the ADNI cohort

Time (days)	Intercept	slope
365	0.02	0.89
730	0.06	1.03
1,095	0.06	1.07
1,460	0.03	1.13
1,825	0.04	1.21

Table S3. Calibration intercept and slope in the NACC cohort

Time (days)	Intercept	slope
365	0.26	0.87
730	0.14	0.82
1,095	-0.01	0.79

Table S4. Results of the ablation analyses

Metric	ARN-Sig	Native-space	Standard-space	Radiomics	R2SN
C-index	0.80	0.77	0.74	0.76	0.75
AUC (t=1 year) (95% CI)	0.79 (0.75-0.86)	0.76 (0.71-0.84)	0.72 (0.66-0.80)	0.75 (0.69-0.82)	0.73 (0.68-0.81)
AUC (t=2 year) (95% CI)	0.85 (0.82-0.88)	0.82 (0.79-0.87)	0.78 (0.76-0.83)	0.81 (0.78-0.85)	0.79 (0.77-0.84)
AUC (t=3 year) (95% CI)	0.87 (0.85-0.90)	0.84 (0.83-0.88)	0.81 (0.79-0.86)	0.83 (0.81-0.87)	0.82 (0.80-0.86)
AUC (t=4 year) (95% CI)	0.89 (0.87-0.92)	0.86 (0.84-0.90)	0.82 (0.80-0.87)	0.85 (0.82-0.89)	0.83 (0.81-0.87)
AUC (t=5 year) (95% CI)	0.90 (0.89-0.94)	0.87 (0.86-0.92)	0.84 (0.82-0.89)	0.86 (0.84-0.91)	0.85 (0.83-0.90)

Table S5. Clustering scores

Cluster	Silhouette	Log-rank	Block R^2	Final score
k=3	0.09	7.85×10^{-32}	0.19	1.00
k=4	0.07	3.48×10^{-33}	0.22	1.03
k=5	0.07	1.61×10^{-40}	0.24	2.23
k=6	0.06	1.74×10^{-32}	0.21	0.52

Table S6. Detailed ARN-Sig Defining the MCI Subtypes

Groups	Spaces	Hemispheres	Regions	Feature Names
Fronto-Parietal Subtype (FPS)	Standard	Right	Frontal Superior Orbital	Local Efficiency
	Native	Left	Parietal Inferior	Original Firstorder Kurtosis
	Native	Right	Temporal Inferior	Original Firstorder Kurtosis
	Standard	Right	Precuneus	Original Firstorder Energy
	Standard	Right	Precuneus	Original Firstorder Kurtosis
	Native	Right	Frontal Superior	Degree
	Native	Left	Occipital Middle	Original Shape LeastAxisLength
Amygdala-Olfactory Subtype (AOS)	Native	Left	Amygdala	Firstorder Range
	Standard	Left	Amygdala	Firstorder Skewness
	Native	Right	Olfactory	Firstorder Skewness
Parahippocampal-Temporal Subtype (PTS)	Native	Right	ParaHippocampal	Original Shape LeastAxisLength
	Native	Left	Temporal Middle	Original GLRLM RunEntropy
	Native	Left	Temporal Pole Middle	Original Shape Maximum2DDiameterSlice
	Native	Left	Temporal Inferior	Original Shape LeastAxisLength
	Native	Left	Temporal Inferior	Original GLDM LargeDependenceEmphasis
	Native	Left	Temporal Inferior	Original Firstorder Kurtosis
Ventral Temporal Subtype (VTS)	Standard	Left	Fusiform	Efficiency
	Standard	Right	Temporal Pole Middle	Local Efficiency
	Native	Left	Fusiform	Original Firstorder Range
Orbitofrontal-Limbic Subtype (OLS)	Standard	Right	Hippocampus	Original Firstorder 10Percentile
	Native	Right	Hippocampus	Original Firstorder Kurtosis
	Standard	Right	Amygdala	Original Firstorder Skewness
	Native	Right	Hippocampus	Original Firstorder Energy
	Native	Right	Rectus	Clustering

Note: Efficiency, Local Efficiency, Clustering, and Degree were classified as R2SNs features, whereas the other features were radiomics features.

Table S7. Absolute conversion rates by subtype in the external validation cohort (ADNI)

Time (days)	FPS	AOS	PTS	VTS	OLS
365	0.29	0.20	0.37	0.14	0.09
730	0.61	0.42	0.74	0.23	0.15
1095	0.78	0.47	0.83	0.33	0.16
1460	0.89	0.58	0.88	0.36	0.16
1825	0.91	0.58	0.89	0.38	0.16
2190	0.93	0.62	0.90	0.38	0.16

Table S8. Absolute conversion rates by subtype in the external validation cohort (NACC)

Time (days)	FPS	AOS	PTS	VTS	OLS
365	0.17	0.08	0.23	0.04	0.03
730	0.50	0.29	0.47	0.16	0.09
1095	0.71	0.41	0.59	0.28	0.10
1460	0.73	0.50	0.65	0.31	0.17

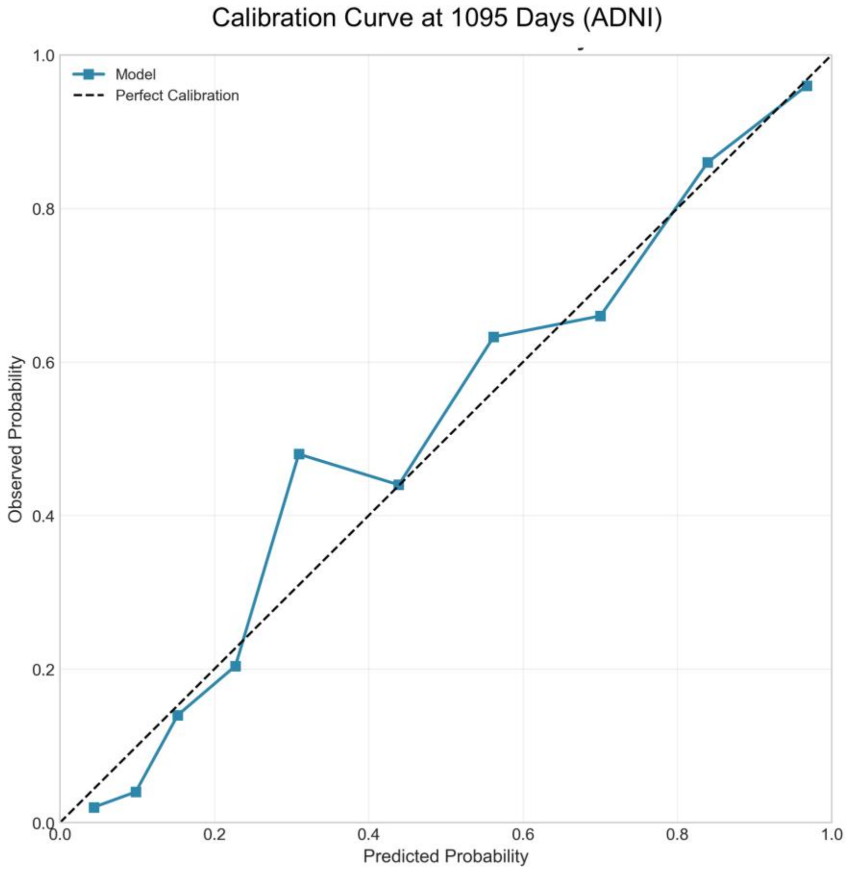

Figure S1. Calibration curve at 1,095 days (ADNI)

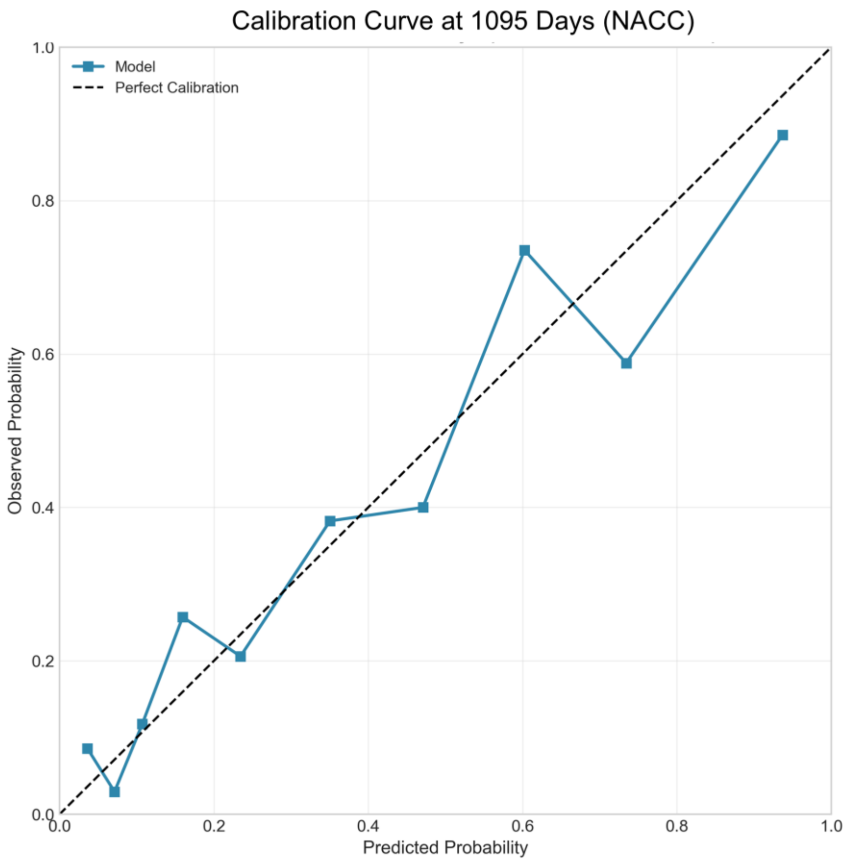

Figure S2. Calibration curve at 1,095 days (NACC)
